# The emerging role of honeysuckle flower in inflammatory bowel disease

**DOI:** 10.3389/fnut.2025.1525675

**Published:** 2025-03-28

**Authors:** Peter Muro, Caihong Jing, Zhihan Zhao, Tao Jin, Fei Mao

**Affiliations:** ^1^Department of Laboratory Medicine, The Affiliated People's Hospital, Jiangsu University, Zhenjiang, Jiangsu, China; ^2^The People's Hospital of Danyang, Affiliated Danyang Hospital of Nantong University, Zhenjiang, Jiangsu, China; ^3^Department of Gastrointestinal and Endoscopy, The Affiliated Yixing Hospital of Jiangsu University, Yixing, China

**Keywords:** inflammatory bowel disease, antioxidant, honeysuckle flower, Traditional Chinese Medicine, *Lonicera japonica*

## Abstract

Crohn’s disease (CD) and ulcerative colitis (UC), referred to as inflammatory bowel disease (IBD), pose considerable challenges in treatment because they are chronic conditions that easily relapse. The occurrence of IBD continues to rise in developing countries. Nonetheless, the existing therapies for IBD have limitations and fail to address the needs of the patients thoroughly. There is an increasing need for new, safe, and highly effective alternative medications for IBD patients. Traditional Chinese Medicine (TCM) is employed in drug development and disease management due to its wide-range of biological activities, minimal toxicity, and limited side effects. Extensive research has shown that certain TCM exhibits significant therapeutic benefits for IBD treatments. Honeysuckle (*Lonicera japonica*) was used in TCM research and clinical settings for the treatment of IBD. Bioactive metabolites in *L. japonica,* such as luteolin, quercetin, cyanidin, chlorogenic acid (CGA), caffeic acid (CA), and saponin, exhibit significant therapeutic benefits for managing IBD. The honeysuckle flower is a potential candidate in the treatment of IBD due to its anti-inflammatory, immune system-regulating, and antioxidant qualities. This paper reviews the metabolites of the honeysuckle flower as a candidate for the treatment of IBD. It discusses the fundamental mechanism of *L. japonica* and the potential of its bioactive metabolites in the prevention and treatment of IBD.

## 1 Introduction

Inflammatory Bowel Disease (IBD) refers to a persistent inflammation in the digestive system, including ulcerative colitis (UC) and Crohn’s disease (CD) (1). IBD is a chronic autoimmune disorder marked by an irregular immune response that targets the gastrointestinal tract, leading to inflammation and tissue damage (2). This places IBD among other autoimmune diseases, such as rheumatoid arthritis, lupus, and multiple sclerosis, where the immune system erroneously attacks the body’s own tissues. These diseases cause substantial morbidity and are difficult to manage effectively, creating an urgent demand for alternative treatment options. The majority of UC lesions occur in the rectal and colonic regions. Initial symptoms include abdominal pain, rectal bleeding, weight loss, and vomiting. CD can manifest in any area of the digestive system, leading to symptoms like stomach pain, diarrhea, bowel blockages, fever, malnutrition, and other signs (3). In addition, CD tends to be chronic, repetitive, and rarely curable (4). Today, several countries and regions continue to face the challenge of IBD, with developed areas experiencing a higher frequency. IBD affects around 1.6 million individuals in the US and up to 2 million in Europe, with an increasing rate of occurrence in developing regions like Asia, Africa, South America, and Eastern Europe (5, 6). Globally, there has been a persistent rise in its occurrence, posing a significant challenge to healthcare systems worldwide (7). Although the pathogenesis of IBD is complex and uncertain, numerous studies confirm that genetics, environmental factors, diet, intestinal integrity, and immune function contribute to its development (4). Genetic factors play a significant role in IBD development, particularly in individuals with a family history of IBD, increasing their vulnerability to the condition (8). According to epidemiological research, environmental factors are significant in the development of UC and CD. These factors throughout life can influence the gut microbiota, thereby contributing to the pathogenesis of IBD (9). Chronic disturbances in gut microbiota can cause long-lasting inflammation and damage the gut lining and barrier (10). This causes the immune system to promptly become active, disrupting the balance of effector and regulatory cells within the intestinal lining. This imbalance contributes to the symptoms observed in IBD (11).

Currently, several treatments exist for IBD, including aminosalicylic acid drugs, corticosteroids, immunomodulators, biological agents, stem cell transplants, fecal microbiota transplants, helminth therapy, and surgery (12, 13). However, current therapies are inadequate for all patients and often lead to severe side effects. The ineffectiveness of certain medications can worsen inflammation and intestinal harm in individuals with IBD (14). For example, aminosalicylic acid preparations, commonly prescribed for early and intermediate-stage IBD treatment, have notable long-term side effects (15). Besides, low patient adherence contributes to a significant recurrence rate of IBD. Similarly, long-term corticosteroid usage may increase mortality risk, especially among older patients who become more dependent on these drugs (16). Patients suffering from IBD require new and efficient medications urgently. This demands the development of cost-effective, safer, and higher anti-inflammatory drugs to address the shortcomings of existing treatments.

One such approach is investigating Traditional Chinese Medicine (TCM) in the treatment of IBD. In TCM, treating conditions such as IBD focuses on restoring the body’s vital energy, or Qi, and balancing the relationship between Yin and Yang (17). According to TCM principles, IBD is commonly linked to a disruption in the balance of internal heat and cold within the body, where an excess of “damp-heat” affects the gastrointestinal tract (18). The treatment is designed to eliminate heat, detoxify the body, and balance the immune system. Research indicates that TCM can effectively regulate the Th17/Treg balance, which is essential in the development of IBD (19). Over the past decade, there has been a significant rise in research on TCM for IBD, with China producing the highest number of publications (20). The ongoing research emphasizes clinical trials, fundamental pharmacology, and animal studies while also exploring emerging trends in network pharmacology and the gut microbiome. A study in Taiwan found that 37% of IBD patients used TCM, primarily herbal remedies, indicating that Western medicine alone may not fully address their medical needs (21). *Lonicera japonica (L. japonica),* or honeysuckle flower, is widely recognized in TCM for its ability to clear heat, detoxify, and alleviate inflammation (22). It is commonly used to treat diseases associated with internal heat, such as infections and inflammatory conditions. The pharmacological properties of *L. japonica*, such as its anti-inflammatory, antioxidant, and immune-regulating effects, correspond with its traditional uses in TCM. These characteristics make it a promising option for managing IBD in both traditional and modern therapeutic contexts.

*L. japonica,* also commonly referred to as Japanese honeysuckle, Jin Yinhua, or Ren Dong, is a member of the *caprifoliaceae* family. This perennial deciduous shrub originates from East Asia and has spread to regions including Argentina, Brazil, Mexico, Australia, New Zealand, and the United States (23). *L. japonica* has gathered interest for its bioactive metabolites, which exhibit antioxidant and anti-inflammatory effects. The Japanese honeysuckle, in particular, is the primary type used in treating IBD. This species is revered for its various bioactive metabolites, such as luteolin, quercetin, chlorogenic acid, caffeic acid, cyanidin, and saponins, which are used in the treatment of IBD. More than 300 chemical metabolites have been isolated and identified from *L. japonica*, and the significant compositions are phenolic acids, essential oils, flavones, saponins, and iridoids (24–26). These metabolites can improve and treat experimental IBD through multiple pathways. Therefore, the progress of these novel therapeutic agents for the prevention and treatment of IBD is of significance. This review aims to summarize research on the potential of honeysuckle flowers in managing and treating IBD.

## 2 Components of honeysuckle flower used in IBD treatment

*L. japonica* has gained attention for its potential therapeutic benefits in the treatment of IBD. *L. japonica’s* key compounds include flavonoids, phenolic acids, saponins, polysaccharides, and organic acids. Understanding its components and effects is vital in developing potential clinical treatments for IBD.

### 2.1 Flavonoids

Flavonoids are secondary metabolites commonly found in natural plants, including *L. japonica* (27). According to pharmacological studies, flavonoids derived from *L. japonica* offer health benefits in preventing cancer, diabetes, cardiovascular diseases, liver damage, and cerebrovascular disease (28, 29). Based on their structural characteristics, flavonoids can be categorized into groups such as flavones, isoflavones, flavanols, flavanones, anthocyanins, and others (30). To date, approximately 52 flavonoids have been identified in *L. japonica*. These consist primarily of flavonols (12 types) and flavones (36 types), with the majority being glycosides (25). The main flavonols include rutin, quercetin, isoquercitrin, astragalin, and quercetin 3-O-hexoside, among others (31). The primary flavones comprise cynaroside, luteolin, chrysoeriol 7-O-neohesperidoside, chrysoeriol 7-O-glucoside, lonicerin, tricin, and others (31). Recently, research highlighted quercetin, luteolin, and cyanidin as the main flavonoids derived from honeysuckle flowers, and they are being studied for their effects on IBD treatment.

**Figure 1:**
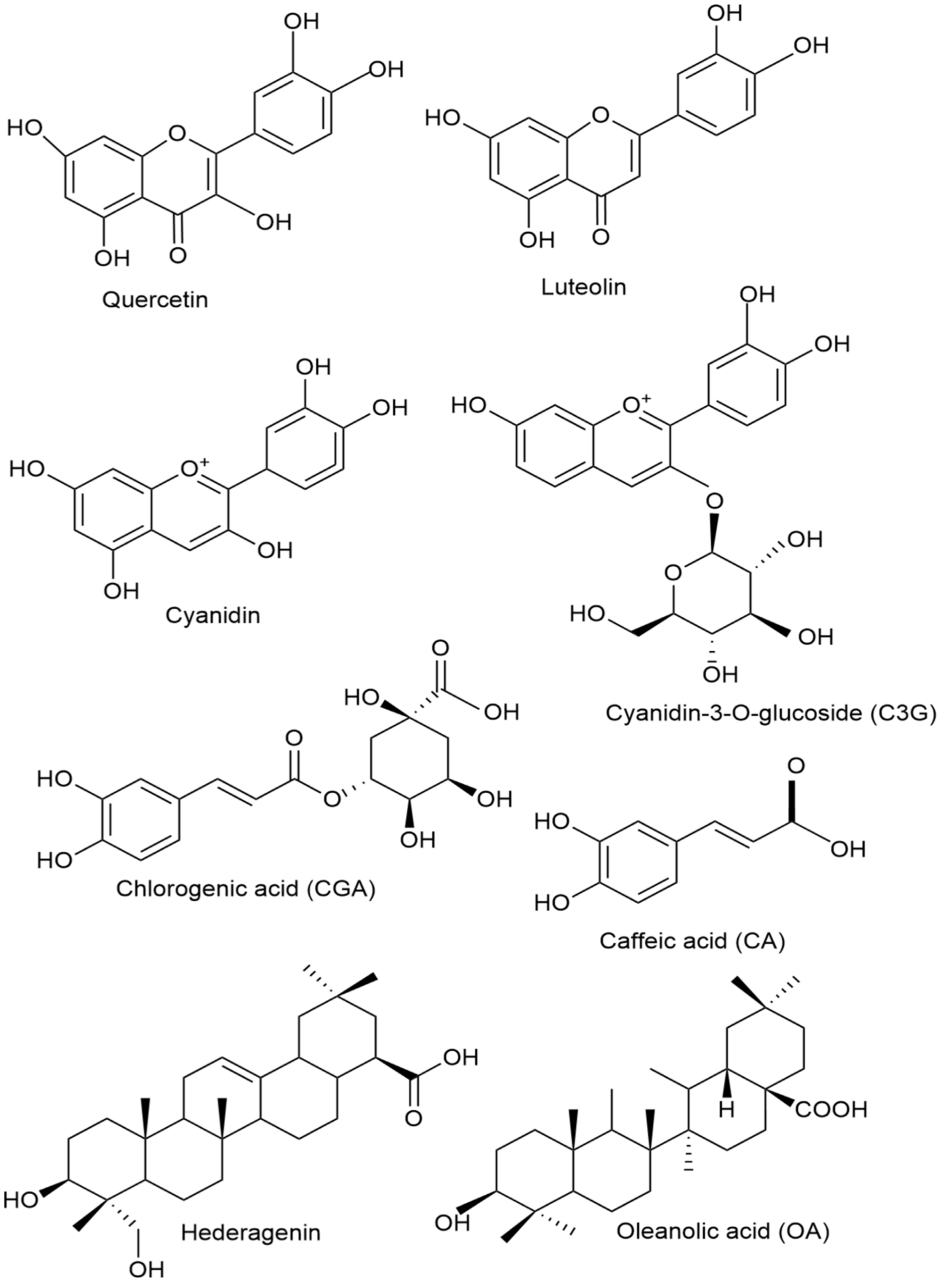
Chemical structures of the key honeysuckle flower bioactive components found in *L. japonica* used in IBD treatment.

Quercetin (3,3,4,5,7-pentahydroxyflavone) is a secondary metabolite found abundantly in plants and is a common constituent in the human diet (32). Quercetin is a flavonoid belonging to the flavonol group that is widely found in polyphenolic compounds in nature. It is typically found in its glycosylated form, where it is bonded with sugar molecules (33). Quercetin is one of the main bioactive metabolites of *L. japonica* used in preclinical studies for the treatment and prevention of IBD. A recent study highlighted the therapeutic efficacy of polyphenols, including curcumin, epigallocatechin gallate (EGCG), resveratrol, and quercetin, in the management of IBD by modulating critical inflammatory pathways such as NF-κB and JAK/STAT (34). This study shows that quercetin and other polyphenols mentioned have therapeutic benefits in IBD treatment by regulating cytokine synthesis and activating the immune cells. However, clinical studies are required to validate their safety and efficacy. The chemical structure of quercetin is depicted in Figure 1.

**Figure 2:**
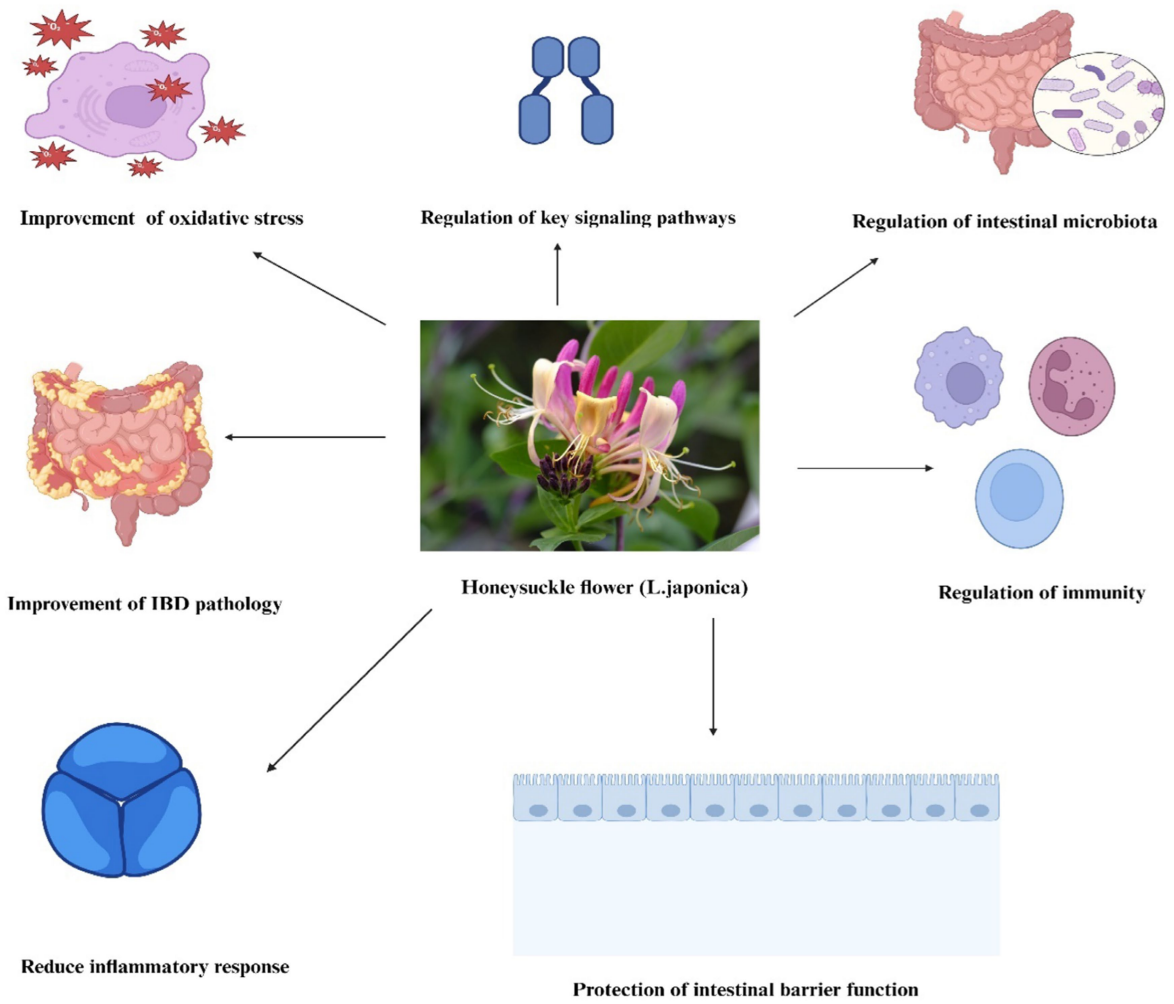
Mechanisms of action of *L. japonica* in managing IBD*. L. japonica* mitigates colitis by (1) regulation of intestinal microbiota, (2) regulation of immunity, (3) protection of intestinal barrier function, (4) improvement of IBD pathology, (5) reducing inflammatory responses, (6) improvement of oxidative stress, and (7) regulation of key signaling pathways. This multifaceted approach helps manage inflammation, protect gut health, and support overall immune function in IBD models.

Luteolin (3,4,5,7-tetrahydroxy flavone), another key bioactive metabolite of *L. japonica,* is commonly found in foods like onions, celery, red peppers, and grapes. Luteolin is a flavonoid belonging to the flavones group. It exhibits a broad spectrum of biological activities, including anti-inflammatory and anticancer effects (35). A recent study developed copper ion-luteolin nano-complexes revealing notable antioxidant and anti-inflammatory properties, indicating their potential as therapeutic agents for IBD by modulating the inflammatory microenvironment, regulating intestinal microbiota, and activating essential oxidative stress pathways, including Nrf2/HO-1 and NF-κB (36). The chemical structure of luteolin is depicted in Figure 2. Furthermore, cyanidin is another key flavonoid used in the IBD model for the prevention and treatment of the disease. Cyanidin and its glycosides are part of the anthocyanin group, a large family of water-soluble plant compounds that contribute to the vibrant red, orange, and blue colors of fruits and flowers (37). Cyanidin-3-O-glucoside (C3G), the most abundant anthocyanin (ACN) in edible fruits, has been linked to several bioactivities, including anti-inflammatory, neuroprotective, antibacterial, antiviral, anti-thrombotic, and epigenetic properties. As a dietary metabolite, cyanidins may offer potential health benefits for humans (38). The chemical structure of cyanidin and its major type, cyanidin-3-O-glucoside, are depicted in Figure 1.

Numerous studies on the IBD animal model also use flavonoids and their metabolite for managing and treating IBD. For instance, citrus-derived flavonoids can modulate IBD by lessening inflammation and preventing intestinal muscle contractions, which notably ameliorates the pathological state in experimental UC rats (39). The fruit of *lycium barbarum* is abundant in flavonoids, including anthocyanins. Researchers investigating the effects of anthocyanins from *Lycium barbarum* on DSS-induced UC in mice discovered that these metabolites alleviated colitis symptoms by influencing three key areas: inflammatory factors, tight junction proteins (TJP), and intestinal microbiota (40). In the same way, water-soluble isoflavones derived from soybeans reduced colitis symptoms in a mouse model by potentially inhibiting inflammation through the modulation of the NF-κB pathway (41). In addition, recent research indicates that luteolin, a major metabolite derived from anthocyanins, could be a promising treatment for UC. It works by mitigating UC symptoms, improving the integrity of the intestinal barrier, and decreasing the production of pro-inflammatory cytokines. These effects are mainly achieved through the inhibition of IKKα/*β* and the suppression of NF-κB signaling pathways (42). These two recent review articles systematically detailed flavonoids as therapeutic agents for managing IBD (43, 44). Hence, flavonoids are highly beneficial in treating acute or chronic intestinal inflammation through various mechanisms, such as shielding against oxidative stress, maintaining the integrity of the epithelial barrier, and exerting immunomodulatory effects in the gut (45). Table 1, summarizes the recent *in vivo* and vitro research progress of flavonoids of *L. japonica*. These studies demonstrate that *L. japonica* flavonoid bioactive metabolites can be used as a potent agent in the treatment of IBD.

**Table 1:** *In vivo* and *in vitro* therapeutic effects of flavonoids of honeysuckle flower on experimental IBD.

No.	Name	Optimal dose (/Kg body weight)	Model of study	Mechanism of action	Ref.
1	Luteolin	20 and 50 mg	DSS-induced C57BL/6 mice.	Reduced inflammation, and oxidative stress, decreasing iNOS, TNF-α, IL-6, and MDA, while increasing SOD, CAT, and Nrf2 targets.	(93)
		50 mg	DSS-induced C57BL/6 mice.	Reduces inflammation, apoptosis, and autophagy in colitis by activating ERK signaling.	(230)
2	Quercetin	10 and 50 mg	Pathogen-free Kunming mice, using IMC cells.	Reduced inflammation and pyroptosis, inhibiting the TLR4/NF-κB/NLRP3 pathway, enhancing cell migration, and protecting tissue structure.	(231)
3	Cyanidin	64.5 mg	TNBS-induced colitis model in BALB/c mice.	Improve clinical symptoms, inhibit myeloperoxidase and cytokines, and protect intestinal barriers in colitis mice and Caco-2 cells.	(101)
4	Cyanidin-3-O-Glucoside	8 mg	DSS-induced in rats.	Significantly improved stool consistency, reduced bleeding, and decreased ileum and colon inflammation. in	(232)
5	Lonicerin	3, 10, and 30 mg	C57BL/6 mice.	Enhancing autophagy, promoting NLRP3 degradation via EZH2 histone methylation, and disrupting NLRP3 inflammasome assembly dose-dependently.	(233)

### 2.2 Phenolic acids

*L. japonica* contains over (49 types) of phenolic acids, primarily consisting of derivatives of chlorogenic acid (CGA) and cinnamic acid (46, 47). A total of 27 CGAs have been isolated and identified from *L. japonica*. These include chlorogenic acid (CGA), neochlorogenic acid (NGC), isochlorogenic acid A (ICA A), isochlorogenic acid B, and isochlorogenic acid C (48, 49). To note, 16 cinnamic acid derivatives, including caffeic acid (CA), 1-O-caffeoylquinic acid, trans-cinnamic acid, trans-ferulic acid, and caffeic acid methyl ester, among others, have been extracted and identified from *L. japonica* (31). CGA and CA are the two most extensively researched metabolites in *L. japonica*, and they have been proven to have potent anti-inflammatory, antioxidant, and antibacterial properties in preventing and managing IBD. CGA is one of the most abundant phenolic acids and is naturally present in green coffee extracts and tea. As an important biologically active dietary polyphenol, CGA exhibits several beneficial and therapeutic effects, including antioxidant, antibacterial, hepatoprotective, and cardioprotective, among others (50). The chemical structure of CGA is depicted in Figure 1. Similarly, CA is a compound derived from plants and classified as a hydroxycinnamic acid, containing both phenolic and acrylic functional groups (51). CA possesses a wide range of beneficial properties, including antioxidant, immunomodulatory, antimicrobial, neuroprotective, anti-anxiolytic, antiproliferative, and anti-inflammatory activities (52). The chemical structure of CA is depicted in Figure 1.

Numerous studies have shown the effects of phenolic acids in managing and treating IBD. For example, it has been documented that dietary phenolic acids alleviate intestinal inflammation by altering gut microbiota composition and regulating macrophage activation. The study enlightens how CGA, CA, ferulic, and ellagic acids affect gut microbiota and macrophages in IBD. Findings indicate that CGA reduces M1 macrophage polarization, while ferulic acid’s effects are neutrophil-dependent, and CA and ellagic acids depend on gut microbiota (53). In the same way, a recent study on *casearia sylvestris* var. *lingua* identified the bioactive metabolites found in *L. japonica,* such as CA, ellagic acid, and quercetin (54). These substances have shown notable anti-inflammatory properties and potential therapeutic benefits in UC models, suggesting their potential for use in managing IBD. It is worth mentioning that CGA significantly mitigates inflammation in IL-10 knockout mice, an IBD model, by increasing CD4^+^/CD8^+^ T cell ratios in Peyer’s patches and mesenteric lymph nodes. Notably, CGA also reduces the expression of inflammatory mediators such as iNOS, IL-1β, and TNF-*α*, demonstrating potential therapeutic benefits for IBD (55).

Moreover, a standardized polyphenol-rich extract from *cynara cardunculus* L. leaves (CCLE) prevents TNF-α-induced NF-κB pathway activation and overexpression of IL-8 and COX-2 in caco-2 cells, enhancing antioxidant power via the Nrf2 pathway. Therefore, CCLE from food-waste leaves shows potential for preventing and treating IBD (56). In addition, the intervention of a newly synthesized caffeic acid phenethyl ester (CAPE) derivative, FA-97, has alleviated DSS-induced colitis by reducing oxidative stress and inflammation, enhancing antioxidant capacity, and activating the Nrf2/HO-1 pathway, as documented (57). FA-97 not only alleviates DSS-induced colitis but also improves normal epithelial barrier function (58). In another study, CAPE reveals reputable anti-inflammatory properties. The study shows that CAPE protects against and treats IBD *in vitro* and dextran sulfate sodium (DSS)-induced UC mouse models, specifically by reducing myeloperoxidases and proinflammatory cytokines, thereby ameliorating NLRP3 inflammasome activity (59). Similarly, another *in vivo* study examined the effects of CAPE using a DSS-induced UC model. The administration of 10 mg/kg of CAPE significantly reduced inflammation markers, including myeloperoxidase activity and cytokine production. This resulted in improved colon length and reduced spleen weight, which indicated a decrease in inflammatory cytokines and cellular adhesion molecules (60). Hence, phenolic acid derivatives of *L. japonica* can be potential candidates for IBD therapy. Table 2 summarizes the recent *in vivo* and vitro research advancements on the phenolic acid of *L. japonica*. These studies show that the phenolic acid bioactive metabolites in *L. japonica* can serve as an effective treatment for IBD.

**Table 2:** *In vivo* and *in vitro* therapeutic effects of phenolic acid and triterpenoid saponins of honeysuckle flower on experimental IBD.

No.	Name	Optimal dose (/Kg body weight)	Model of study	Mechanism of action	Ref.
1	Chlorogenic acid (CGA)	250 and 500 mg	DSS-induced colitis mice.	Ameliorates DSS-induced colitis in mice via Nrf-2/HO-1 pathway activation, enhancing antioxidative and anti-inflammatory responses.	(172)
		40 mg	DSS-induced acute colitis in BALB/c mice.	Downregulating miR-155 expression and inactivating the NF-κB/NLRP3 inflammasome pathway in macrophages.	(199)
2	Isochologenic acid A (ICG-A)	25 and 50 mg	DSS-induced colitis mice.	Reducing neutrophil infiltration and pro-inflammatory cytokines, and inhibiting STAT3/NF-кB pathways.	(113)
3	Caffeic acid	50 mg	DSS-induced experimental UC.	Reduces UC severity and improves mucosal inflammation, serum indexes, and cytokine secretion, targeting macrophage activation.	(109)
	Caffeic acid	251 mg	DSS-induced colitis mice.	Reduced colonic inflammation, lowered pro-inflammatory cytokines, boosted antioxidants, activated Nrf-2/HO-1, and balanced gut microbiome in mice.	(160)
4	Ginsenoside Rg1	200 mg	DSS-induced acute colitis in C57BL/6 mice.	Alleviation of acute UC by modulating balance gut microbiota and microbial tryptophan metabolism.	(131)
5	Ginsenoside Rh2	50 mg	DSS-induced acute colitis in C57BL/6 J mice.	Suppressing STAT3/miR-214 activation, indicating potential for UC treatment.	(234)
6	Ginsenoside Rb1	40 mg	DSS-induced acute colitis in C57BL/6 mice.	Alleviates colitis by normalizing Hrd1 expression, reducing ER stress markers, and decreasing fas-related apoptosis in mouse models.	(235)

### 2.3 Triterpenoid saponins

Triterpenoid saponins are natural substances recognized for their wide range of pharmacological activities, particularly their anti-inflammatory properties, which could make them promising candidates for IBD treatment (61). Most saponins found in *L. japonica* are of the oleanane and hederagenin types (62). Hederagenin, a triterpenoid saponin prevalent in honeysuckle and various medicinal plants, acts as the aglycone for numerous saponins (63). It acts as the aglycone for many saponins and demonstrates a variety of pharmacological properties, including anti-tumor, anti-inflammatory, and anti-viral effects (64). Oleanic acid is another triterpenoid saponin found in honeysuckle (65). Oleanolic acid is a triterpenoid substance known for its hepatoprotective, anti-inflammatory, and anticancer effects. It is present in a variety of plants, such as rosemary, melissa, peppermint, sage, and ginseng (66, 67). In addition, lonicerosides, such as loniceroside A and B, are specific saponins isolated from *L. japonica* (68). These compounds contribute to *L. japonica’s* antioxidant, anti-inflammatory, and antimicrobial properties (31). The chemical structure of hederagenin and oleanic acid are depicted in the Figure 1.

Numerous studies show the impact of triterpenoid saponins in the treatment of IBD. For example, a recent study reveals the therapeutic potential of oleanane-type triterpenoid saponins, particularly 23-methyl-3,28-bisdesmosidic saponins, in ameliorating UC by enhancing the intestinal epithelial cell barrier and modulating intestinal flora (69). Notably, the saponins, particularly 23-methyl-3,28-bisdesmosidic oleanane-type saponins, were found to inhibit the TNFα-NFκB-MLCK axis of the colon in UC mice, highlighting their critical role in UC treatment. In the same way, this previous study highlights the significant anti-inflammatory and immune-modulating properties of saponins, including lonicerosides, natural glycosides found in remedial plants such as honeysuckle, which play a crucial role in the treatment of IBD and other intestinal-inflammatory conditions by suppressing inflammation, promoting intestinal barrier repair, and maintaining gut flora diversity (70). Natural products and herbal medicines, including hederagenin, have shown efficacy in treating IBD in experimental models and clinical trials (71). Table 2 summarizes the recent *in vivo* and vitro research advancements on the triterpenoid saponin of honeysuckle flowers. These studies show that the saponins bioactive metabolites in *L. japonica* can serve as an effective treatment for IBD.

### 2.4 Essential oils

The essential oils extracted from honeysuckle flowers contribute to its medicinal properties. Essential oils are a key bioactive metabolite of *L. japonica*, primarily consisting of acids, aldehydes, alcohols, ketones, and their esters (72). These oils include compounds such as geraniol and linalool, which have anti-inflammatory and antimicrobial qualities that may help manage and treat IBD (73). This study (74) shows that coated pellets and mini-tablets containing CIN-102, a linalool extract, and a novel antibacterial blend were developed for colitis treatment, aiming to optimize antibiotic concentration in the colon, significantly reducing pathogenic bacteria and improving clinical outcomes in colitis-induced mice. Similarly, geraniol, known as (Trans)-3,7-Dimethyl-2,6-octadien-1-ol, exhibits notable anti-inflammatory and antimicrobial effects (75). It shows promise as a treatment for colitis, as both oral and enema administration in mice has reduced inflammation and corrected dysbiosis, underscoring its potential as an effective medication for managing intestinal inflammation and dysbiosis. It is worth mentioning that geraniol has potential as a drug candidate due to its antitumor, anti-inflammatory, antioxidant, antimicrobial, hepatoprotective, cardioprotective, and neuroprotective properties (76). In a randomized, double-blind study involving 56 patients with irritable bowel syndrome (IBS), a low-absorbable geraniol supplement significantly alleviated IBS symptom severity and improved responder rates. Geraniol also caused notable alterations in gut microbiota, particularly affecting oscillospira and faecalibacterium populations (77). Table 3 summarizes the recent *in vivo* and vitro research advancements on the essential oil of *L. japonica.* These studies have shown that the essential oil bioactive metabolites in *L. japonica* can serve as an effective treatment for IBD.

**Table 3:** *In vivo* and *in vitro* therapeutic effects of essential oil or terpenoids and polysaccharides of honeysuckle flower on experimental IBD and clinical study.

No.	Name	Optimal dose (/Kg body weight)	Model of study	Mechanism of action	Ref.
1	Geraniol	100 mg	DSS-induced UC in mice.	Reduced pro-inflammatory cytokines, inhibited NF-κB signaling, and restored antioxidant parameters.	(236)
		90 mg	Double-Blind Randomized Clinical Trial.	Reduced IBS symptoms severity and altered gut microbiota composition, especially in the irritable bowel syndrome (IBS) mixed subtype, without affecting proinflammatory cytokines.	(77)
		8 mg	A pilot study.	Increases beneficial gut bacteria like Faecalibacterium, reduces IBS symptoms, and lowers inflammatory marker MIP-1β.	(205)
2	Linalool	200 mg	Acetic acid (AA)-induced UC in rats.	Reduced MDA, IL-1β, IL-6, COX-2, NF-κB, and TNF-α while increasing Nrf-2 and CAT in UC rats.	(237)
3	*L. japonica* thunb polysaccharides	50, 100, and 150 mg	DSS-induced UC in mice.	Improved weight, organ index, serum cytokines, SIgA, NK cell activity, and intestinal probiotics in colitis mice.	(86)
		50, 100, and 150 mg	Male Balb/c mice.	Enhances immune response in CTX-treated mice by increasing lymphocyte proliferation, macrophage phagocytosis, and NK cell activity.	(238)

### 2.5 Polysaccharides

Natural polysaccharides possess strong bioactive properties, particularly notable for their anti-inflammatory effects. In recent years, natural polysaccharides have gathered significant attention due to their high efficacy, safety, and easy availability. Polysaccharides are among the primary active metabolites of *L. japonica*, which have been isolated and characterized in earlier studies (78). Honeysuckle flowers *(L. japonica)* possess three polysaccharides with anti-inflammatory properties (LJP-1, LJP-2, LJP-3), which hold promise for applications in functional foods and healthcare products (79).

Numerous studies have documented natural polysaccharide’s therapeutic and beneficial effects on IBD (80). In an *in vitro* study, polysaccharide extracts from *L. japonica* demonstrated significant DPPH-scavenging activity, ABTS+ scavenging activity, hydroxyl radical scavenging activity, superoxide radical scavenging activity, and inhibitory solid effects on erythrocyte hemolysis induced by hydrogen peroxide (H₂O₂) (81). Similarly, polysaccharide extracts from *L. japonica* have the potential to protect mouse cardiomyocytes from hydrogen peroxide-induced damage by enhancing the activities of catalase (CAT), glutathione peroxidase (GSH-Px), and superoxide dismutase (SOD), while simultaneously reducing the production of reactive oxygen species (ROS) (82). Yet again, a study conducted in live organisms demonstrated that raw polysaccharides derived from *L. japonica* could reduce liver oxidative harm in diabetic rats induced by streptozotocin (STZ). This reduction was proved by lower levels of alanine aminotransferase (ALT), aspartate aminotransferase (AST), and gamma-glutamyl transpeptidase (GGT) in the blood, alongside increased levels of CAT, SOD, and GSH in the liver (83). Polysaccharides significantly contribute to the antioxidant capabilities of *L. japonica.* Table 3, summarizes the recent *in vivo* and vitro research advancements on the polysaccharides of honeysuckle flowers. These studies have shown that the polysaccharides bioactive metabolites in *L. japonica* can serve as an effective treatment for IBD.

## 3 Honeysuckle flower as a promising candidate for IBD treatment

Despite remarkable advancements in modern medicine, significant challenges persist in the treatment of IBD, a major health concern for people globally. The cause of IBD is attributed to a multifaceted interaction between genetic, environmental, and immune-related factors (84). IBD conventional treatments include aminosalicylates, corticosteroids, immunomodulators, and biological therapies (85). However, these treatments can have significant side effects and are not always effective, leading to an interest in alternative and complementary therapies. One such promising complementary treatment is the use of honeysuckle flower (*L. japonica*), known for its anti-inflammatory and immunomodulatory properties (86). *L. japonica* is rich in numerous bioactive metabolites, such as phenolic acids, flavonoids, saponins, essential oils, iridoids, and polyphenols (31). Primary active components of these bioactive metabolites include luteolin, quercetin, cyanidin, chlorogenic acid (CGA), caffeic acid, saponins, and others, which have anti-inflammatory and antioxidant effects that can be used in IBD treatment (87). Luteolin (3,4,5,7-tetrahydroxyflavone) is a prevalent flavonoid found in celery, honeysuckle, garden bitter melon stems, and other global plants (88). Among the various flavonoids, luteolin stands out for its notable impact on IBD, as demonstrated by numerous experimental IBD models. For instance, this study (89) reported the effects of luteolin on DSS-induced colitis in mice. Luteolin tends to improve colon length/body weight ratio, reduce spleen weight/body weight ratio, increase SOD, decrease MDA and cytokines, and downregulates HMGB1 expression, mitigating colitis via HMGB1-TLR-NF-κB pathway modulation. Further studies reveal that luteolin promotes the transformation of NCR-MNK3 to NCR + MNK3 *in vitro*, enhances IL-22 production, reduces IL-17a and INF-*γ* levels, restores NCR-ILC3/NCR + ILC3 balance, improves intestinal barrier function in DSS-induced UC mice by upregulating ZO-1 and occludin, linked to the notch pathway, suggesting luteolin’s potential in UC treatment (90). Also, luteolin treatment appears to promote beneficial alterations in the gut microbiota, increasing the amounts of lactobacillus, bacteroides, rosebery, and butyricoccus while decreasing DSS-induced elevated ratios of lactobacillus and prevotella (91). A recent study found that administrating luteolin into the peritoneum increased beneficial bacteria that fight inflammation, such as clostridia UCG-014, enterorhabdus, blautia, and the lachnospiraceae *NK4A136* group, and decreased depraved bacteria that cause inflammation, such as turicibacter, streptococcus, staphylococcus, clostridium sensu stricto 1, romboutsia, parasutterella, and escherichia-shigella (92). Notably, luteolin significantly outperformed apigenin or xanthohumol treatment in reducing related physical UC symptoms. Alternatively, luteolin (20 and 50 mg/kg) lowered the levels of inflammatory substances such as iNOS, TNF-*α*, and IL-6. It also reduced the disease activity index (DAI), colon shortening, and histological damage by a significant amount (93). This shows that luteolin may help maintain or restore gut microbial balance, improving health and reducing inflammation. In summary, luteolin is a promising flavonoid found in *L. japonica* that can potentially provide therapeutic benefits for IBD. Nevertheless, additional research on human study is necessary to confirm these results in clinical settings, identify effective dosing strategies, and comprehend the mechanisms by which luteolin affects gastrointestinal health.

Similarly, quercetin is another effective flavonoid bioactive metabolite of *L. japonica*, which has been used in numerous animal model studies in managing IBD. Quercetin is a widely utilized natural flavonoid that is typically found in glycosylated forms, including rutoside (3-rhamnosy-glucosyl quercetin) and quercitrin (3-rhamnosylquercetin) (94). This study reveals that giving oral quercetin doses between 25 and 100 mg/kg over 11-day period is associated with decreased body weight loss, rectal bleeding, and both visible and biochemical intestinal damage in mice (95). The potential mechanism involves quercetin’s antioxidant properties, which may reduce lipid peroxidation and oxidative stress by modulating nitrite and nitrate levels, enhancing GSH levels, and lowering myeloperoxidase (MPO) activity in the colonic mucosa (96). Quercetin can also alleviate experimental colitis by modulating macrophage’s anti-inflammatory and bactericidal activities via an HO-1-dependent pathway, and administering it through diet could help restore intestinal balance and promote healthy gut microbiota as a potential IBD treatment (97). It has been further documented in this murine colitis model study that quercetin-loaded microcapsule-treated mice show significantly higher ABTS radical cation scavenging and ferric-reducing activity, reduced neutrophil influx (MPO activity), edema, and colonic inflammation, decreased levels of pro-inflammatory cytokines IL-1β and IL-33 while maintaining IL-10 levels, and preserved endogenous antioxidants compared to colitis control mice (98). Additionally, a recent study suggests that quercetin has a protective function in the management of IBD. A comprehensive UK Biobank investigation revealed that individuals with elevated dietary quercetin consumption exhibited a markedly diminished risk of acquiring IBD, especially UC (99).

In the same way, a recent prospective cohort study suggests that dietary quercetin may offer promising therapeutic benefits for individuals with IBD (100). The research, comprising 2,293 individuals (764 with CD and 1,529 with UC) from the UK Biobank, examined the correlation between quercetin consumption and adverse results such as enterotomy and overall mortality. Higher quercetin intake participants had a noticeably lower risk of enterotomy and death after a mean follow-up of 9.6 years than those with the lowest consumption. Using Cox proportional hazard models, the study examined the data and found that although with differing degrees of correlation, quercetin had a protective effect in both CD and UC. This provides persuasive evidence for the potential of quercetin as a dietary supplement in IBD management, warranting further research in clinical trials. In summary, quercetin shows significant promise for treating IBD due to its anti-inflammatory and antioxidant properties, as evidenced by multiple studies. Future research should focus on bioavailability and standardized protocols to enhance consistency, clarifying quercetin’s role in IBD management and supporting its use in standard treatments.

Cyanidin, found in honeysuckle, is also another key component of *L. japonica* in the form of cyanidin-3-O-glucoside (C3G), which is the most prevalent anthocyanin (ACN) used in various animal studies for treating IBD (38). C3G is approved for its antioxidative, anti-inflammatory, cytoprotective, and antimicrobial properties, which can be beneficial for managing IBD (38). The administration of C3G at a 200 μmol/kg dosage significantly improved clinical symptoms and histological damage in Trinitrobenzenesulfonic acid (TNBS)-induced colitis mice and ameliorated intestinal barrier destruction in LPS-stimulated Caco-2 cells, as documented (101). C3G suppressed inflammatory cytokines and nitric oxide production, indicating its potential as a preventive agent for IBD. In LPS-stimulated HUVECs and acute respiratory distress syndrome mouse models, C3G inhibited pro-inflammatory cytokines, reduced oxidative markers, and suppressed NF-κB and MAPK pathways, mitigating inflammation and oxidative injuries (102). Notably, increased free fatty acids (FFAs) in obesity contribute to systemic lipotoxicity and inflammation, affecting IBD progression, while C3G, which induces Nrf2-regulated proteins, mitigates these effects by reversing palmitic acid-induced inflammation and redox imbalance in Caco-2 cells via NF-κB and Nrf2 pathways, suggesting their potential as therapeutic agents for intestinal inflammation (103). Interestingly, a systematic review analyzed human intervention studies, evaluating the presence of anthocyanins like C3G in plasma and urine using high-throughput techniques (104). The study, which included 10 clinical trials, found that C3G was most frequently present in human urine (58.06%) and plasma (69.49%), fulfilling key criteria for a biomarker, including dose–response, time response, stability, and analytical performance. The molecule’s positive predictive value was 74% in plasma and 61.7% in urine, indicating its potential as a biomarker of berry intake in healthy humans, thus suggesting its relevance in health applications such as IBD or cancer prevention. In the same way, this recent pilot trial demonstrated the efficacy of Infliximab (IFX) therapy for IBD (105). The study included 47 IBD patients who were given either a low-anthocyanin red fruit tea or a high-anthocyanin purple maize supplement. The trial discovered that the purple maize supplement increased IFX-mediated disease remission, especially in CD patients. This shows that C3G could be a promising bioactive molecule for IBD treatment and prevention when used with traditional medications such as IFX.

Moreover, among the different phenolic acids of *L. japonica*, chlorogenic acid (CGA) and caffeic acid (CA) are particularly significant for their pronounced effects on IBD, as shown by various experimental IBD models. For example, this study (106) evaluates the impact of dietary CGA on colon damage and bacterial profiles in a DSS-induced colitis mouse model. It shows that CGA reduced disease activity, myeloperoxidase activity, tumor necrosis factor-*α* levels, colon shortening, and histological scores while improving microbial diversity, remarkably increasing lactobacillus abundance. These findings suggest CGA will likely maintain intestinal health and mitigate colitis by reducing pro-inflammatory cytokines and restoring microbial diversity. CGA also shows potential in treating IBD by targeting the TLR4 signaling pathway, which is crucial in inflammatory responses and cell processes (107). On the same note, CGA can reduce MPO expression levels by inhibiting the TLR4-mediated, PI3K/Akt, and NF-κB pathways, leading to decreased NEUT infiltration and lower expression of pro-inflammatory cytokines in both DSS-induced colitis and LPS-stimulated RAW 264.7 cells (108). On the other hand, caffeic acid (CA) can significantly reduce mucosal inflammation by improving disease severity, biochemical indexes, mucosal ulcerations, cytokine secretion, and modulating macrophage function and activation, suggesting CA as a potential UC therapy (109). A study looked at how different types of caffeic acid reduced inflammation and discovered that 5-caffeoylquinic acid (5-CQA) at 25 μmoL/L and 10 μmoL/L of caffeic acid significantly decreased pro-inflammatory cytokines (TNF-*α*, IL-1β, and IL-6) while increasing the anti-inflammatory cytokine IL-10 (110). This suggests that 5-CQA could be useful for treating diseases that are caused by inflammation. Similarly, *Ilex rotunda*, a caffeic acid derivative, exhibited notable anti-inflammatory effects by suppressing the production of NO and several pro-inflammatory cytokines, including IL-6, IL-1β, TNF-α, and IL-8, in LPS-stimulated RAW264.7 macrophages and HT-29 colon epithelial cells (111). The study confirmed that *Ilex rotunda* also reduced the expression of inflammatory markers such as inducible nitric oxide synthase (iNOS), cyclooxygenase (COX-2), and phosphorylated ERK1/2, suggesting its potential as a therapeutic candidate for IBD. Interestingly, a recent *in vivo* study also reveals that dietary caffeic acid at a dosage of (80 μg/kg) body weight significantly improved intestinal barrier function and microbiota diversity in piglets experiencing LPS-induced intestinal damage, resulting in an increase in lactobacillus and terrisporobacter and a decrease in romboutsia (112). The findings prove that caffeic acid successfully restores the levels of bile acids and acetate reduced by LPS exposure, implying its capacity to alleviate intestinal injury and enhance gut health in juvenile mice. Yet again, isochlorogenic acid A (ICGA-A), another vital phenolic acid, alleviated symptoms in DSS-induced colitis model mice by reducing inflammation, neutrophil infiltration, and pro-inflammatory cytokine production through controlling STAT3/NF-кB signaling, suggesting ICGA-A as a potential UC treatment (113).

Furthermore, saponins, which are found in honeysuckle flowers, also exhibit potent anti-inflammatory properties, help maintain immune balance, and are frequently used in animal models for treating IBD, as documented (70). In treating IBD, saponins suppress intestinal inflammation, repair intestinal barriers, maintain intestinal flora diversity, and reduce colon cancer incidence rates (70). One of the main saponins used in IBD treatment is ginsenosides, the main components of ginseng, which have demonstrated anti-inflammatory and immunomodulatory effects, suggesting potential in treating and managing IBD (114). Ginsenosides, particularly Rk3, show significant potential in treating UC by reducing inflammation and protecting intestinal barrier function, as documented (115). This study demonstrates that Rk3 administration alleviates symptoms of DSS-induced UC in mice by inhibiting the NLRP3 inflammasome pathway and restoring colonic integrity. Additionally, It documents that oral administration of sanguisorba saponin extract (SSE) for 7 days significantly reduces pro-inflammatory factors and colon damage in a 2.5% DSS-induced UC mouse model, with low-polarity saponins like ZYS-II improving lipid metabolism disorders and normalizing phosphatidylcholine levels, indicating SSE’s potential in UC treatment (116). Again, this recent study documents that saponins from polygonatum sibiricum (PSS) improve UC symptoms such as weight loss, DAI scores, and colon length in male kunming mice induced with DSS. Histopathology and 16S rRNA analysis reveal that PSS restores intestinal microbial richness and diversity, reduces pathogenic bacteria, and increases lactobacillus spp. and muribaculaceae, thereby alleviating colitis symptoms (117).

These findings suggest that *L. japonica* has the potential to be a therapeutic agent or candidate for IBD treatment. Although preclinical studies are promising, clinical evidence in humans is still limited. A few small-scale clinical trials have explored the use of honeysuckle in IBD patients. However, more prominent, well-designed clinical trials are needed to confirm these findings and establish the safety and efficacy of honeysuckle flowers in IBD treatment. *L. japonica* is generally considered safe when used appropriately. Though, further research is needed to fully elucidate the mechanisms by which honeysuckle exerts its therapeutic effects in IBD. Large-scale clinical trials are necessary to determine optimal dosing, safety, and long-term efficacy. Exploring the synergistic impact of honeysuckle flower bioactive metabolites with conventional IBD treatments could provide new avenues for combination therapies.

## 4 Honeysuckle flower extract interactions with standard IBD treatments

*L. japonica* has long been recognized for its therapeutic properties, particularly its anti-inflammatory and antioxidant effects. Recent studies have emphasized its potential in treating IBD, a long-term condition marked by inflammation in the gastrointestinal tract. However, the bioactive metabolites of *L. japonica*, such as flavonoids, phenolic acids, and saponins, could potentially lead to important drug–drug interactions when combined with common IBD treatments like corticosteroids, immunosuppressants, and biologic agents. Understanding how these therapies interact is vital for ensuring both the safety and effectiveness of combined treatment approaches in IBD patients.

Corticosteroids are one of the most commonly utilized classes of drugs in IBD treatment, as they suppress inflammation during outbreaks (118). Honeysuckle’s bioactive metabolites, such as flavonoids, have been proven to have anti-inflammatory activities. Although these substances could potentially enhance the anti-inflammatory effects of corticosteroids, they may also affect the metabolism of corticosteroids. For instance, the cytochrome P450 (CYP450) enzyme system processes many bioactive substances in plants and is responsible for the elimination of many medicines, including corticosteroids (119). As a result, co-administration of honeysuckle flower extracts may inhibit or stimulate these enzymes, affecting corticosteroid pharmacokinetics and potentially resulting in reduced efficacy or increased toxicity. On the other hand, Immunosuppressants, such as azathioprine and methotrexate, are widely used to treat IBD by inhibiting the immune response that causes inflammation (120). The bioactive metabolites of *L. japonica,* particularly flavonoids, have been shown to have minor immunomodulatory effects (26). This raises concerns that the combination of honeysuckle and immunosuppressants may result in unanticipated immune function changes. While some research suggests that TCM honeysuckle may reduce the effectiveness of immunosuppressants by modulating immune pathways (121), others suggest that it may enhance the immunosuppressive effects, increasing the risk of infections or other side effects associated with suppressed immune responses (122).

In the same way, the utilization of biologic medicines, including anti-TNF-*α* monoclonal antibodies such as infliximab, is becoming more prevalent in the management of moderate to severe IBD (123). These medications function by targeting particular proteins implicated in the inflammatory process. Research on the potential interaction between honeysuckle bioactive metabolites and biologics is currently uncommon. Nevertheless, given that biologic medicines are delivered through infusion and metabolized through analogous pathways to corticosteroids and immunosuppressants, there exists a potential for honeysuckle to either augment or diminish their efficacy. Due to the effectiveness of biological medicines and their notable adverse effect profiles, meticulous evaluation is necessary when integrating them with plant-derived substances such as *L. japonica.*

In summary, it’s important to note that the bioactive metabolites of honeysuckle flowers might help treat IBD, but they might also interact with other traditional IBD medicines. Clinical investigations are urgently required to assess the pharmacokinetic and pharmacodynamic interactions between TCM honeysuckle bioactive metabolites and corticosteroids, immunosuppressants, and biologics (124). In the lack of such data, prudence is recommended while integrating honeysuckle with these medications, and healthcare professionals should meticulously observe patients for any adverse reactions or alterations in treatment effectiveness.

## 5 Mechanism of honeysuckle flower repair in IBD

### 5.1 Modulation of intestinal microbiota

The balance of the intestinal microbiota is essential for maintaining human intestinal health. Patients with IBD often exhibit an imbalance in their gut microbiota (125). When the balance of gut bacteria is distressed, the intestine’s immune regulation and protective functions are weakened. This leads to the harming of the intestinal lining and worsens the clinical symptoms in patients with IBD (126). It has been documented that UC patients have increased firmicutes, actinobacteria, bacteroides, and others, while bacteroidetes, bifidobacterium, and several other bacteria are decreased (127). Although it is not clear whether gut microbiota imbalance is a causative factor or a pathogenic consequence of IBD, several honeysuckle flower bioactive metabolites have demonstrated that modulating the gut microbiota can improve clinical symptoms of experimental IBD (128–130). Numerous bioactive metabolites in honeysuckle flower exhibit therapeutic effects in IBD, influencing the intestinal microbiota to varying extents (Figure 2). Ginsenoside Rg1 alleviates DSS-induced colitis in mice by improving colonic injury, inflammation, and gut microbiota composition, mainly through regulating tryptophan metabolism (131). Similarly, *Lonicera rupicola hook (LRH)* flavonoids improved the gut microbiota composition and diversity in DSS-induced UC mice, suggesting potential as a therapeutic agent by modulating the gut microenvironment and suppressing the PI3K/AKT pathway (132). In addition, research has shown that natural polysaccharides (NPs) can help treat IBD by changing the gut microbiota and boosting the production of short-chain fatty acids (SCFAs) (133). This leads to better gut immunity, reduced inflammation, and more substantial gut barriers. In the same way, the intervention of non-starch polysaccharides (NSPs) shows promise in the treatment of IBD through various mechanisms, including anti-inflammation and gut microbiota modulation, necessitating further research for clinical applications (134). In other studies, *laminaria japonica polysaccharides (LJP)* alleviate obesity and improve gut health by reducing inflammation and altering gut microbiota composition, as demonstrated in an animal obesity model (high-fat diet). The high-fat diet model study showed significant benefits of LJP, including weight loss, improved blood serum indicators, and increased probiotic gut bacteria levels (135). In the same way, this recent study reveals that quercetin administration improves colitis symptoms and histopathological alterations by enhancing mucin production, increasing goblet cell numbers, and upregulating tight junction protein expression essential for intestinal integrity (136). The result shows that quercetin helps promote M2 macrophage polarization, decreases inflammatory cytokines, and activates the Nrf2/HO-1 signaling pathway, which reduces oxidative stress and modifies microbial composition to favor beneficial bacteria over harmful ones. Other recent findings reveal quercitin’s significant enhancement of beneficial bacteria such as akkermansia and lactococcus, along with elevated levels of SCFAs like propanoate, isovalerate, and hexanoate. These metabolites correlate with quercitin’s ability to modulate gut microbiota composition and activity, thereby mitigating intestinal inflammation (137).

### 5.2 Regulation of immunity

In the development of IBD, the immune system undergoes alterations due to abnormal reactions of both the innate and adaptive immune systems. In IBD patients and animal studies, there are usually more T-helper 17 (Th17) cells and fewer Treg cells in the blood (138). Recent studies indicate that immunometabolic activities in IBD primarily involve T cells, monocytes, macrophages, dendritic cells, and natural killer cells (139). Immune cells generate inflammatory cytokines that are crucial in managing the inflammatory response. Therefore, focusing on immune cell metabolism might be a promising approach to the treatment of IBD. Numerous studies have shown that honeysuckle flower bioactive metabolites can alleviate IBD symptoms by influencing immune and metabolic activities (Figure 2). For instance, CGA, a bioactive metabolite of honeysuckle flower, can regulate immunity by increasing the ratio of CD4+/CD8+ T cell subsets in Peyer’s patches and mesenteric lymph nodes of IL-10 knockout mice. CGA also reduces the expression levels of inflammatory mediators such as iNOS, IL-1β, and TNF-*α*, attenuating colon inflammation (55). Similarly, a previous study showed that CGA inhibits TNFα- and H_2_O_2_-induced IL-8 production in human intestinal cells (108). In DSS-induced colitis mice, CGA alleviates symptoms and reduces inflammatory markers like colonic macrophage inflammatory protein 2 and IL-1β mRNA expression, suggesting its potential to regulate immune responses in intestinal inflammation (140). Besides, a recent study showed that luteolin enhances innate lymphoid cell (NCR^+^ ILC3) proportion through Notch signaling, regulating immunity in UC by restoring intestinal barrier function and reducing pathology (141). Moreover, luteolin has the potential as a natural regulator of immune system inflammatory signaling processes, offering anti-inflammatory effects by altering pathways such as NF-κB, MAPK, Janus kinase/signal transducer activator of transcription, and inflammasome signaling (142). This implies that focusing on the metabolic activities of immune cells might offer a new strategy for treating IBD.

### 5.3 Protection of intestinal barrier function

The main sign of intestinal barrier dysfunction is the increased permeability of the intestinal lining (143). The two main factors contributing to this damage are the death of intestinal epithelial cells (IEC) and the breakdown of tight junctions between cells. The inner IECs act as a barrier separating the inner lumen from the external environment. Research indicates that apoptosis of IECs in the colon plays a role in the onset of chronic IBD (144). IEC apoptosis plays a crucial role in the development of IBD. When there’s an excess of IEC apoptosis, it can disturb the gut’s defense mechanisms and weaken the intestinal barrier function (145). Numerous studies have proven that *L. japonica’s* bioactive metabolite can inhibit IEC apoptosis through multiple pathways (Figure 2) (146). The key pathways involved include the death receptor pathway, the mitochondrial pathway, the endoplasmic reticulum stress pathway, the MAPK pathway, the NF-κB pathway, and the PI3K/Akt pathway (147). For instance, procyanidin A1 (PCA1) alleviates DSS-induced UC in mice and LPS-stimulated cell models by enhancing cellular immunity. This is demonstrated by reduced proinflammatory cytokines, apoptosis, and histological damage via the AMPK/mTOR/p70S6K pathway (148). Similarly, luteolin, a flavonoid extract of honeysuckle flower, shows significant therapeutic potential in enhancing intestinal barrier function by increasing the abundance of anti-inflammatory microorganisms and reducing pro-inflammatory cytokines in a murine model of UC (92). Another study also reveals that luteolin shows anti-inflammatory impact on intestinal inflammation via the inhibition of the JAK/STAT pathway and the maintenance of tight junction integrity in cytokine-stimulated HT-29 colon epithelial cells, reducing IL-8, COX-2, iNOS expression, and ˙NO overproduction (149). This demonstrates luteolin’s efficacy in modulating inflammatory signaling cascades. Tight junctions facilitate intercellular connections; they enable selective osmotic sealing between neighboring IECs, forming a barrier that safeguards intestinal tissues. Also, they are composed of the cytoplasmic zonula occludens (ZO) protein family, transmembrane proteins such as tricellulin, nectin, occludin, and claudins, as well as cytoskeletal components (17). The disruption of tight junction results in a compromised intestinal immune system and inflammation, which is closely associated with the onset of IBD (150). Similarly, in a DSS-induced colitis model, quercetin restored tight junctions (TJs) in an aryl hydrocarbon receptor (AhR)-dependent manner (151). The effects were significant at doses of 25, 50, and 100 mg/kg *in vivo* over 10 days. These results were supported by *in vitro* studies that showed increases in TJs proteins like ZO-1 and Claudin1 expression in caco-2 cells along with AhR activation. However, using the AhR antagonist CH223191 showed a possible limitation because it reversed the effects seen. This suggests that quercetin’s effectiveness may depend on the integrity of the AhR pathway, which could affect its usefulness in clinical settings. These studies have shown collectively that honeysuckle flower bioactive metabolites help maintain the integrity of the intestinal barrier by preventing apoptosis of IECs and restoring tight junctions.

### 5.4 Improving general IBD pathological symptoms

The clinical signs of IBD include blood in the stool, stomach pain, frequent diarrhea, and loss of weight (152). In the chemical agent-induced IBD model in mice or rats, pathological conditions typically include colonic inflammation, weight loss, diarrhea, bloody stools, and colon shortening, closely resembling the clinical manifestations of human IBD, with the DAI score, averaging weight loss, fecal condition, and blood-stool scores, which are utilized for the pathological assessment of IBD severity (153). Studies have shown that the use of *L. japonica’s* bioactive metabolite has ameliorative and therapeutic effects on experimental IBD and improves its pathological symptoms (Figure 2). For example, buthanol (BuOH) extracts of *L. japonica* improved IBD pathology in DSS-induced colitis mice by significantly reducing crypt injury, inflammation scores, and serum amyloid and MPO levels compared to 5-ASA (154). It also decreased IL-6 levels in LPS-stimulated HT-29 cells, suggesting the potential for IBD prevention in humans. In the same way, the bioactive metabolite gallic acid, found in *L. japonica,* notably decreased DAI and colonic shortening in UC mice and mitigated pathological damage to colonic tissue (155). The intervention of intraperitoneal injection of flavonoids, particularly luteolin and xanthohumol, significantly improved IBD pathology, reduced DAI, and was revealed as a potentially effective IBD treatment by modulating gut microbiota composition and reducing pro-inflammatory cytokines in a murine model of UC (92). Recently, this study showed that using a DSS-induced zebrafish IBD model revealed *chrysanthemum morifolium* extracts significantly improved IBD pathology, while *L. japonicas* bioactive metabolites, including Isochlorogenic acid C, Isochlorogenic acid A (ICA A), other phenolic acids, flavonoids, and polysaccharides, reduced IL-1β, IL-8, and MMP9 expressions, demonstrating notable anti-inflammatory and antioxidant effects (156). Natural compounds such as astragalus polysaccharide and quercetin are promising for improving IBD pathology by providing effective treatments with fewer side effects and tackling issues related to drug resistance and disease recurrence (157). Moreover, honeysuckle metabolites significantly improve UC pathology by reducing pro-inflammatory cytokines, increasing SCFAs, and restoring intestinal ecological balance (22). Hence, these studies show that honeysuckle could be a candidate for the treatment of IBD.

### 5.5 Reducing inflammatory responses

Inflammation is a bodily process triggered by the immune system in reaction to injury, infection, or stress (158). The main therapeutic benefit of honeysuckle flowers in experimental IBD is the enhancement of the colonic inflammatory response. Excessive inflammatory reactions and the overproduction of pro-inflammatory factors worsen colitis symptoms in IBD. This inflammation results from an excessive immune cell response within the body, including an overactive Th1 or Th2 T-cell response (159). Excessive immune cell activity leads to significant alterations in cytokines, involving the pro-inflammatory cytokines IL-1, IL-2, IL-6, IL-12, IL-18, IFN-*γ*, and TNF-*α*, as well as the anti-inflammatory cytokines IL-4, IL-5, IL-10, and IL-13. Numerous bioactive compounds of honeysuckle flower modulate inflammatory cytokines through multiple pathways to attenuate the IBD inflammatory response (Figure 2). Caffeic acid (CA) was reported to significantly inhibit the generation of inflammatory cytokines (TNF-*α*, IL-6, IL-12, and IL-1*β*) in the colon and disrupt the infiltration and activity of mononuclear macrophages in the mucosa, mediastinal lymph nodes, and spleens of DSS-induced mice (160). A dose of 50 mg/kg of chlorogenic acid (CGA) inhibited inflammation (TNF-α, IL-1β, and IL-6) in the colons of mice treated with indomethacin (161). In addition, CGA notably elevated the CD4+/CD8+ T cell subset ratio in Peyer’s patches and mesenteric lymph nodes while decreasing the expression levels of iNOS, TNF-α, and IL-1β (55). CGA also exhibits anti-inflammatory properties by inhibiting IL-8 production in caco-2 cells stimulated by TNFα and H2O2, as well as attenuating DSS-induced colitis in mice, indicating its potential as a dietary supplement for IBD (140). On the other hand, quercetin also suppresses the inflammatory response in IBD by inducing miR-369-3p, which reduces C/EBP-β, TNF-α, and IL6 levels in LPS-stimulated dendritic cells, demonstrating its potential as a therapeutic agent, as documented (162). Another study showed that a colon-specific quercetin delivery system (COS-CaP-QT) exhibited anti-inflammatory properties by activating the notch pathway (163). This activation helped regulate T helper 2 cells and group 3 innate lymphoid cells, contributing to the remodeling of the inflammatory microenvironment and easing colitis symptoms. Also, this recent *in vitro* investigation utilizing caco-2 cells, varied dosages of Q (1, 10, and 100 μM) were administered to alleviate IL-1β-induced permeability disturbances via inflammatory signaling pathways, notably NF-κB p65, ERK1/2, MLCK, and p-MLC (164). At non-toxic dosages (Q1 and Q10 μM), quercetin substantially reduced IL-1β-driven increases in paracellular permeability and downregulated MLCK gene transcription, highlighting its therapeutic potential for intestinal barrier preservation. However, a significant restriction was seen at the Q100 μM level, which led to a decrease in transepithelial electrical resistance (TER) after 24 h. This suggests that greater doses may affect cellular integrity, therefore warranting careful dose assessment in clinical applications.

### 5.6 Improving oxidative stress

Numerous studies have revealed a strong link between the onset of IBD and the impairment of the antioxidant system. In the intestines of individuals with IBD, high levels of oxidative stress result in lipid peroxidation, DNA damage, cell apoptosis, and inflammation (165). Overproduction of reactive oxygen species (ROS), such as superoxide anion radicals, hydrogen peroxide, and hydroxyl radicals, can compromise the intestinal mucosal barrier. This disruption occurs by changing the levels of oxidative enzymes and pro-inflammatory cytokines, leading to an inflammatory response and worsening IBD (166). In the mouse model of IBD, the concentrations of nitric oxide (NO), myeloperoxidase (MPO), superoxide dismutase (SOD), malondialdehyde (MDA), and catalase (CAT) are changed in the colonic tissues, leading to a disruption in intestinal function (167). *L. japonica* has effective antioxidant activities, especially polyphenols and flavonoids (Figure 2). For example, the polyphenolic compound procyanidin B2, considered a bioactive metabolite of the honeysuckle flower, was found to have an ameliorative effect on the symptoms of IBD in experimental mice by inhibiting oxidative stress in colonic tissues via Nrf2/ARE signaling, which promotes the repair of intestinal damage (168). Similarly, Flos lonicerae flavonoids (hyperoside, lonicerin, and luteolin) significantly reduced oxidative stress in a rat model of UC; pretreatment with these flavonoids lowered serum levels of oxidative markers like SOD and MDA, demonstrating their potent antioxidative effects (169). Notably, Intervention with the flavonoid luteolin administration in a mouse model of experimental colitis significantly reduced oxidative stress by decreasing MDA levels and increasing SOD and catalase CAT activities; additionally, luteolin elevated nuclear factor-erythroid 2-related factor 2 (Nrf2) levels and its downstream targets, heme oxygenase-1 (HO-1) and NADP(H): quinone oxidoreductase 1 (NQO1), showcasing its potent antioxidative effects (93). In addition, this recent study documents that honeysuckle bioactive metabolites, like polyphenols, show promising effects on IBD treatment due to their ROS scavenging properties and ability to enhance antioxidant defenses (170). These natural compounds can inhibit pro-oxidative enzymes, thereby reducing inflammation and aiding in the management of IBD through pathways like NF-κB and Nrf2. Quercetin also significantly improved oxidative stress by reversing H_2_O_2_-induced cell damage, decreasing ROS and apoptosis ratio, and upregulating GCLC transcription, which increased intracellular glutathione levels, ultimately alleviating oxidative stress in a caco2 cell model and DSS-induced colitis (171). Besides, CGA supplementation effectively ameliorates DSS-induced colitis by activating the Nrf-2/HO-1 pathway, which suppresses oxidative stress and inflammatory responses and promotes gut barrier function (172). Hence, improving oxidative stress is crucial for treating IBD, where ROS plays a significant role.

### 5.7 Regulation of key signaling pathways

Abnormal signaling pathway activation causes an unregulated inflammatory response in individuals with IBD (Figure 2) (173). Several studies have demonstrated that various signaling pathways play crucial roles in the onset and progression of IBD. As discussed below, TCMs, such as honeysuckle flowers (*L. japonica*), can alleviate and treat IBD by influencing multiple signaling pathways.

#### 5.7.1 NF-κB signaling pathway

**Figure 3:**
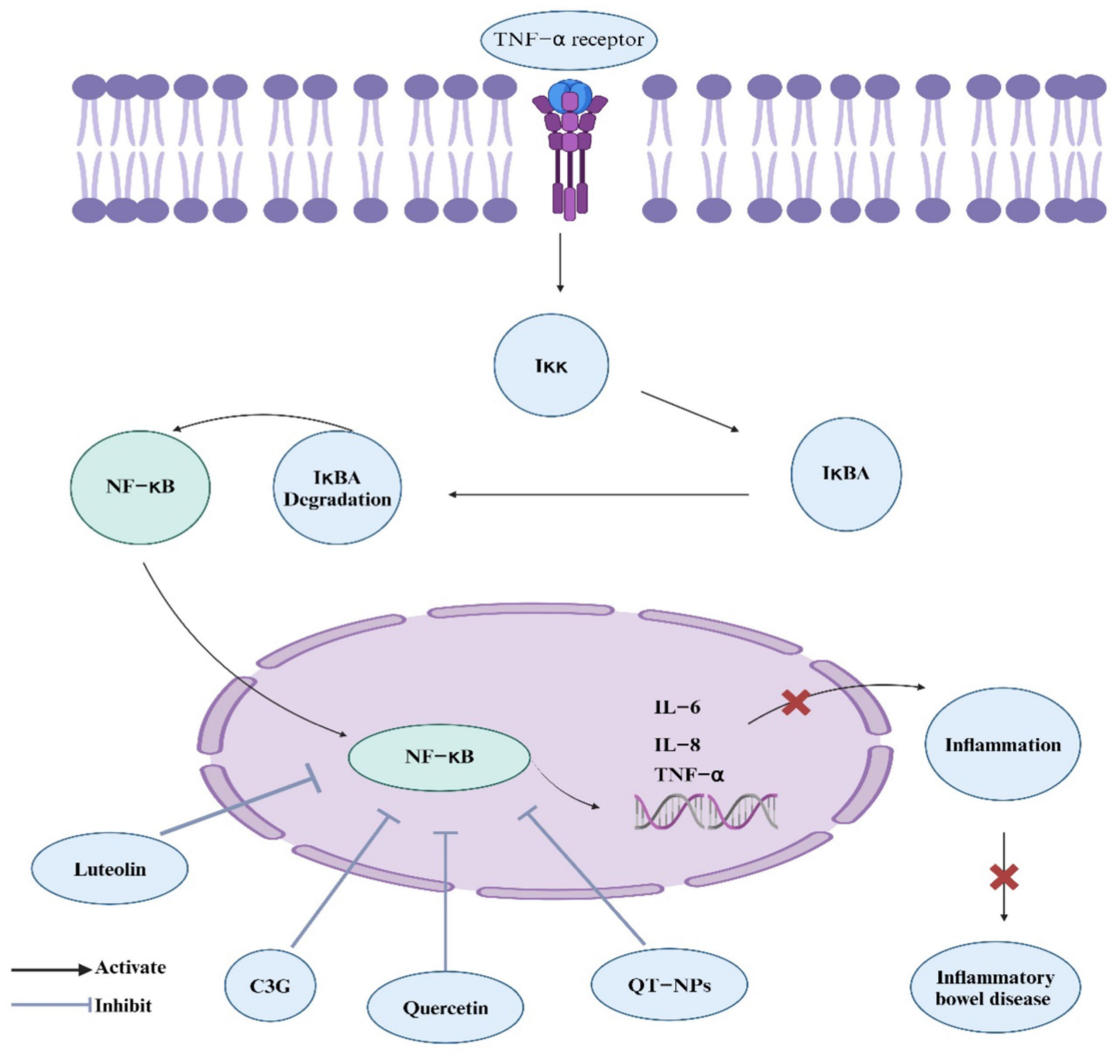
*L. japonica* regulation of NF-κB signaling pathway. The figure depicts the regulatory mechanisms of the NF-κB signaling pathway, which plays a pivotal role in inflammation and immune responses associated with IBD. Bioactive components from *L. japonica,* such as luteolin, cyanidin-3-glucoside (C3G), and quercetin, inhibit NF-κB activation. This inhibition leads to a reduction in inflammatory responses and alleviation of IBD symptoms.

NF-κB is an essential intracellular nuclear transcription factor that plays a significant role in the body’s inflammatory and immune reactions. It also regulates apoptosis and stress responses. Excessive activation of NF-κB is strongly linked to numerous human diseases related to inflammation (174). In the model of IBD induced by chemical agents, the NF-κB signaling pathway is activated and significantly influences the progression of IBD (175). Targeting the NF-κB signaling pathway with drugs might be a potential method for treating IBD. Research on *L. japonica* metabolites has mainly demonstrated their regulatory impact on the NF-κB signaling pathway, which could help alleviate IBD. Notably, luteolin, a flavonoid from *L. japonica*, inhibits NF-κB signaling by preventing NF-κB activation, IκB degradation, and luciferase activity in PMA plus A23187-induced mast cell activation. This suppression reduces inflammatory cytokines TNF-*α*, IL-8, IL-6, and GM-CSF, decreasing intracellular Ca^2+^ levels and ERK 1/2 and JNK 1/2 activation, highlighting luteolin’s role in managing inflammatory diseases (176). Flos Lonicera flavonoids, which contain hyperoside, lonicerin, and luteolin, significantly attenuate UC in rats by reducing oxidative and inflammatory biomarkers. The flavonoids significantly inhibit the NF-κB pathway, highlighting their potential therapeutic role in UC through antioxidative and anti-inflammatory mechanisms (56). In addition, quercetin, a flavonoid in *L. japonica*, suppresses LPS-induced inflammation in RIMVECs by inhibiting TLR4 and NF-κB p65 expression, reducing IκB-*α* degradation and attenuating ERK, JNK, and STAT phosphorylation, while the MAPK p38 pathway remains uninvolved, highlighting quercetin’s potential in targeting the NF-κB signaling pathway to mitigate inflammation (177). Yet again, this recent study highlights that quercetin-loaded nanoparticles (QT-NPs) also exhibit promising therapeutic effects in IBD by modulating the NF-κB pathway (178). The treatment notably reduces the expression of pro-inflammatory cytokines and key enzymes such as COX-2 and iNOS, contributing to the alleviation of oxidative stress and inflammation in colonic tissues, which are crucial factors in the pathogenesis of IBD. Moreover, In IBD, excessive activation of the NF-κB pathway, triggered by TNF-α, leads to intestinal inflammation and dysfunction, but pretreatment with C3G inhibits this pathway and improves cellular redox status in caco-2 cells (179). These studies suggest their potential role in managing and treating IBD through modulation of the NF-κb pathway (Figure 3).

#### 5.7.2 MAPK signaling pathway

**Figure 4:**
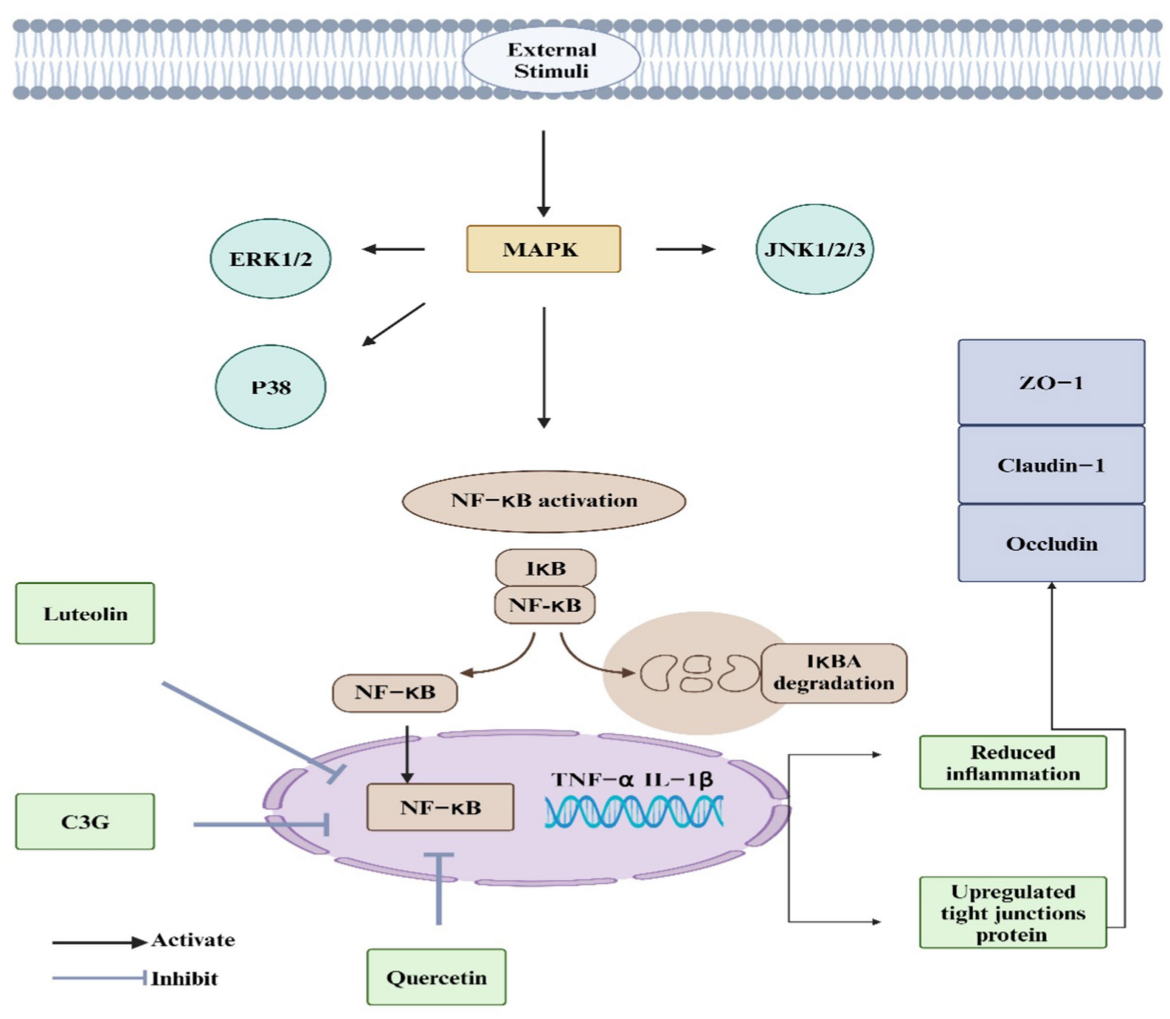
*L. japonica* regulation of MAPK signaling. The MAPK signaling pathway plays a pivotal role in the pathogenesis of IBD. Upon activation by external stimuli, this pathway influences key kinases, including extracellular signal-regulated kinases 1 and 2 (ERK1/2), c-Jun N-terminal kinases 1, 2, and 3 (JNK1/2/3), and p38 MAPK. These kinases, in turn, activate the nuclear factor kappa-light-chain-enhancer of activated B cells (NF-κB). Bioactive compounds derived from *L. japonica*, such as luteolin, cyanidin-3-glucoside (C3G), and quercetin, effectively inhibit NF-κB activation. This inhibition leads to the upregulation of tight junction proteins, which enhances the integrity of the intestinal barrier, thereby reducing inflammation and alleviating IBD symptoms.

The MAPK signaling pathway is a well-known inflammatory signaling route that responds to external stimuli. Activation of this pathway primarily affects extracellular signal-regulated kinases 1 and 2 (ERK1/2), C-Jun N-terminal kinases 1, 2, and 3 (JNK1/2/3), and p38 (Figure 4) (180). MAPK is a signaling molecule that activates NF-κB and is crucial in inflammatory diseases. Research revealed that proteins related to the MAPK signaling pathway were significantly elevated in the colonic tissues of a DSS-induced IBD mouse model, indicating that the MAPK pathway is activated in this model of IBD (181). Using *L. japonica* can improve IBD by inhibiting the activation of the MAPK signaling pathway. For example, the total flavone of *Abelmoschus manihot* L. Medic (TFA), including quercetin, alleviated TNBS-induced colitis in mice by inhibiting the NF-κB and MAPK signaling pathways. Quercetin derivatives in TFA, such as quercetin-3-O-robinobioside, contributed to reduced cytokine levels and inflammation. This suggests TFA’s potential in treating CD through MAPK pathway modulation (182). Similarly, quercetin ameliorates intestinal barrier disruption and inflammation in acute necrotizing pancreatitis (ANP) by inhibiting the TLR4/MyD88/p38 MAPK signaling pathway and ERS activation (183). This intervention reduces pancreatic and ileal pathological damage, intestinal tight junction disruption, and inflammation markers like IL-1β, TNFα, and IL-17A. Quercetin thus shows protective effects against ANP-induced damage. Not only that, this study indicates that luteolin ameliorates ethanol-induced intestinal barrier damage in a caco-2 cell model by upregulating tight junction proteins (ZO-1, occludin, claudin-1), suppressing MLC phosphorylation, MLCK activation, NF-κB nuclear translocation, MAPK phosphorylation, and enhancing ARE and Nrf2 nuclear translocation, thereby alleviating oxidative stress and tight junction dysfunction (184). Hence, *L. japonica* bioactive metabolite can be used as potential candidates for managing IBD through this pathway.

#### 5.7.3 JAK/STAT signaling pathway

The JAK/STAT signaling pathway is a crucial downstream pathway activated by cytokines involving JAK1, JAK2, JAK3, TYK2, and STAT proteins. This pathway significantly manages mucosal damage, inflammation, and immune response regulation (185). Phytochemicals like curcumin and quercetin, particularly honeysuckle bioactive metabolites, target multiple JAK–STAT sites, showing anti-inflammatory, antitumor, and cardiovascular benefits (186). CGA, the primary bioactive metabolite of honeysuckle flower, suppresses IL-6-stimulated proliferation of fibroblast-like synoviocytes (RSC-364) by inducing apoptosis. This effect is mediated by the inhibition of the JAK–STAT signaling pathway, reducing the expression of p-STAT3, JAK1, and gp130, as well as the NF-κB pathway (187). As previously mentioned, luteolin, a bioactive metabolite in honeysuckle flower, exhibits anti-inflammatory properties by inhibiting the JAK/STAT signaling pathway in cytokine-stimulated HT-29 colon epithelial cells. This inhibition reduces IL-8 production, COX-2, and iNOS expression, suggesting luteolin’s potential as a therapeutic strategy for IBD (118). It is worth mentioning that luteolin also improves intestinal epithelial barrier integrity in IBD by promoting tight junction protein expression and suppressing the STAT3 signaling pathway via SHP-1 regulation (188). Similarly, this recent study shows that luteolin-7-O-glucoside (Lut-7-G) treatment reduced colon inflammation, weight loss, and disease activity in DSS-induced UC in mice by modulating the JAK1/STAT6/SOCS1 pathway, significantly decreasing IL-6, IL-1β, IL-18, and TNF-*α* levels, as well as JAK1 and STAT6 expression, while upregulating SOCS1 expression, demonstrating its potential in UC treatment (189). Hence, the JAK–STAT signaling pathway is critical in the biological effects of cytokines involved in CD and UC. Therapies targeting JAKs, like tofacitinib, show promise by affecting multiple cytokine-dependent pathways, providing new treatment options for patients unresponsive to traditional cytokine-targeting therapies (190).

#### 5.7.4 PI3K and TLRs

Phosphatidylinositol-3-kinase (PI3K) plays a role in modulating cell surface receptor signaling and is involved in leukocyte activation, growth, and proliferation. Notably, in this study (191), the role of PI3K in IBD is highlighted by its involvement in human mucosal smooth muscle cells (M-SMCs). Virus-induced cellular stress increases hyaluronan (HA) deposition, promoting leukocyte attachment. This response is PI3K/Akt-dependent, as shown by the inhibition of HA-mediated leukocyte adhesion by PI3K inhibitor LY294002. The transplantation of hypoxia-inducible factor (HIF)-1α-overexpressing mesenchymal stem cells (HIF-MSC) effectively treated IBD by regulating macrophage polarization via the phosphatidylinositol-3-kinase (PI3K) pathway (192). HIF-MSCs upregulated PI3K-*γ* downstream targets, enhancing anti-inflammatory macrophage responses. PI3K-γ inhibition diminished these effects, highlighting PI3K’s critical role in HIF-MSC-mediated IBD therapy. As previously mentioned, the recent study on LRH honeysuckle flavonoids identified their beneficial effects on UC; LRH flavonoids suppress the PI3K/AKT pathway, reduce inflammation, and improve the gut microbiome, showing significant potential for UC treatment through their anti-inflammatory and antioxidant properties (132). Toll-like receptors (TLRs) are a crucial category of protein molecules that participate in nonspecific immunity by detecting unusual molecules and initiating the body’s immune response. TLRs are essential for maintaining the balance of the intestinal mucosa. TLRs are decisive for maintaining the balance of the intestinal lining. Conversely, experimental models of IBD show a decrease in TLR expression, particularly TLR4 (193). Numerous bioactive metabolites of honeysuckle flower are involved in TLR pathway changes. For example, CGA effectively protects the intestinal barrier by blocking the TLR4/NF-κB pathway. It downregulates CD14 and p65, preventing phospho-p65 nuclear translocation, and inhibits pro-inflammatory cytokines such as TNF-*α*, IL-1β, and IL-6, restoring tight-junction integrity. Thus, CGA shows potential for anti-inflammatory therapies by targeting TLR signaling (194). Similarly, quinic acid, a honeysuckle bioactive metabolite, demonstrate therapeutic potential in treating UC by targeting the TLR4, NF-κB-inducible nitric oxide synthase (INOS), and nitric oxide (NO) signaling pathways (195). As previously mentioned, luteolin also shows promising effects by significantly alleviating DSS-induced colitis in mice by reducing HMGB1 mRNA and protein levels and downregulating the HMGB1-TLR-NF-κB signaling pathway. This leads to an increase in colon length/body weight ratio, decreased spleen weight/body weight ratio, elevated SOD levels, and reduced MDA levels in serum and intestine, thereby highlighting its therapeutic potential for IBD (89). Targeting the TLR4/NF-κB signaling pathway might be an effective strategy to alleviate intestinal inflammation, with polyphenol phytochemicals that contain honeysuckle showing noticeable alleviative effects on intestinal inflammation by acting on this pathway (196). This suggests the potential for treating CD and UC through PI3K and TLRs pathway modulation, providing an effective approach to treating IBD in future clinical research applications.

#### 5.7.5 NLRP3 inflammatory vesicles

Inflammatory vesicles are crucial elements of the body’s innate immune system and significantly influence the onset and advancement of IBD. Of all the pattern recognition receptors, NLRP3 inflammasomes are the most strongly linked to IBD and have been the primary focus of research and investigation by researchers (197). NLRP3 inflammasomes, a vital component of the innate immune system, are found in increased levels in individuals with IBD as well as in animal models used for IBD research. These inflammasomes remain persistently active in response to various triggers throughout the progression of the disease (198). Numerous bioactive metabolites found in *L. japonica* can reduce the clinical symptoms in experimental IBD models by preventing the activation of NLRP3 inflammasomes, thus aiding in the improvement and treatment of IBD. For example, a honeysuckle flavonoid, CGA, effectively represses the nucleotide-binding domain-like receptor protein 3 (NLRP3) inflammasome, a key player in UC pathogenesis. Using LPS/ATP-induced RAW264.7 cells and DSS-induced colitis in mice, CGA was shown to reduce NLRP3 inflammasome-related proteins and miR-155 expression, suggesting a potential therapeutic strategy for UC (199). In the same way, quercetin, a honeysuckle flavonoid, inhibits IL-1β secretion by both the NLRP3 inflammasomes and AIM2 inflammasomes in a dose-dependent manner, reducing apoptosis-associated speck-like protein containing a CARD (ASC) speck formation and oligomerization. This autophagy-independent effect highlights quercetin’s potential as a therapeutic candidate for inflammatory diseases such as IBD and Kawasaki disease vasculitis by preventing NLRP3 inflammasome activation (200). Similarly, in this study, quercetin-loaded silk fibroin nanoparticles (QSFN) demonstrate significant anti-inflammatory effects in a mouse model of colitis by inhibiting the NLRP3 inflammasome and reducing pro-inflammatory cytokines like IL-1β, suggesting QSFN as a promising treatment for IBD, where quercetin alone was ineffective due to poor bioavailability and stability (201). Hence, Targeting NLRP3 inflammatory vesicles with honeysuckle metabolite can reduce inflammation, improve safety, and offer novel therapeutic options for IBD treatment.

#### 5.7.6 PPARγ

Peroxisome proliferator-activated receptors (PPARs) are nuclear receptors found abundantly in the colon, which are involved in numerous physiological processes, regulate gene expression, and are crucial in the onset of inflammation (202). PPARs exist in three distinct isoforms: PPARα, PPARβ, and PPAR*γ*. Of these, PPARγ is particularly important in the context of IBD and is targeted in the development and treatment of IBD medications (203). Recent studies have shown that several *L. japonicas* metabolite can have ameliorative effects on IBD by activating and modulating PPAR*γ*. For instance, Luteolin, a honeysuckle flavonoid, acts as a partial agonist/antagonist on PPARγ, inhibiting some target genes and adipogenesis while activating GLUT4 like rosiglitazone; in IBD, it uniquely binds to PPARγ without stabilizing the activation helix, providing significant anti-inflammatory effects without promoting adipocyte differentiation, making it a safer alternative to thiazolidinediones (TZDs) for treating other inflammatory conditions such as type II diabetes (204). Similarly, luteolin also ameliorates irinotecan-induced inflammation and oxidative stress in Caco-2 cells by upregulating PPARγ, HO-1, and SOD and downregulating IL-1β and iNOS; however, its protective effect is lost when PPARγ is downregulated, except for IL-1β, indicating luteolin’s PPARγ-dependent mechanism in IBD (205). A study demonstrates that C3G, a honeysuckle flavonoid, activates PPAR-γ and Nrf2 pathways, reducing oxidative stress and inflammation in HT-29 cells, suggesting their potential as nutraceuticals for managing IBD (206). C3G outperforms 5-aminosalicylic acid in reducing cytokine-induced inflammation, highlighting its effectiveness in IBD treatment.

## 6 Effects of honeysuckle flower on other intestinal digestive disorders

### 6.1 Spontaneous colorectal cancer

Colorectal cancer (CRC) is a significant global health concern, often exacerbated by unhealthy lifestyles prevalent in Western countries (207). Various natural compounds, including those found in *L. japonica,* have shown promise in combating this disease. For instance, C3G, the primary anthocyanin in many plants, has demonstrated efficacy in overcoming oxaliplatin (OXA) resistance in CRC. C3G treatment in OXA-resistant CRC cell lines reversed epithelial-mesenchymal transition (EMT), a key mechanism behind chemoresistance. This reversal led to enhanced apoptosis, reduced cell migration, and restoration of the epithelial phenotype, suggesting C3G’s potential in CRC treatment (208). Likewise, luteolin has also shown significant therapeutic potential candidates in CRC. In studies involving azoxymethane (AOM)-induced CRC in Balb/C mice, luteolin treatment effectively reduced the expressions of iNOS and COX-2, demonstrating its anti-inflammatory properties. These findings highlight luteolin’s potential as a novel, side-effect-free drug for CRC (209). Simultaneously, quercetin, a prominent plant flavonol, exhibits significant antitumor effects in CRC. Studies using an AOM/DSS-induced CRC mouse model revealed that quercetin treatment markedly reduced tumor size and count, restored leukocyte counts, and decreased oxidative stress markers. These results emphasize quercetin’s anti-inflammatory and antioxidant properties (210). Similarly, quercetin promotes gasdermin D (GSDMD)-mediated pyroptosis in colon cancer cells by upregulating NEK7, enhancing NLRP3 inflammasome assembly, and GSDMD cleavage. This mechanism suggests quercetin as a therapeutic target for CRC (211). Additionally, quercetin enhances the efficacy of ginsenoside Rg3 (Rg3) against CRC by eliciting ROS and converting the immunosuppressive tumor microenvironment. Combined with Anti-PD-L1, this approach significantly prolongs animal survival (212). Furthermore, CGA, also demonstrates anti-cancer properties against CRC by inducing dose-dependent cytotoxicity, cell cycle arrest, and apoptosis in HT-29 and HEK-293 cells through the modulation of apoptosis-related genes and ROS levels (213). Besides, linalool, an essential oil in *L. japonica*, has also been investigated for its anticancer mechanisms in CRC, as it induces apoptosis in human colon cancer cells by generating cancer-specific oxidative stress, and *in vivo*, high-dose linalool significantly reduced tumor weight, suggesting its potential in CRC therapy (214). Thus, the therapeutic potential of natural metabolites, including those from *L. japonica*, in treating colorectal cancer looks promising. These metabolites exhibit various mechanisms, such as reversing chemoresistance, reducing inflammation, inducing apoptosis, and modulating gut microbiota, highlighting their potential as novel, effective treatments for CRC.

### 6.2 Colitis-associated cancer

Colitis-associated cancer (CAC) is a significant complication arising from prolonged inflammation in the intestinal tract, typically seen in patients with IBD (215). Recent studies suggest that dietary polyphenols, found in various plants, including honeysuckle flower, can modulate the gut microbiota, thereby alleviating colitis and reducing the risk of CAC (216). These polyphenols increase the abundance of beneficial bacteria such as Lactobacillus and Bifidobacterium while decreasing pro-inflammatory microbes. The interactions between these gut microbiota and polyphenol metabolites are believed to contribute to the protective effects of polyphenols against colitis and CAC. Additionally, ginsenoside compound K, another honeysuckle-derived metabolite, has shown potential anti-colorectal cancer (CRC) effects by modulating gut microbiota, notably upregulating *akkermansia muciniphila*, and significantly suppressing tumor growth and restoring gut microbiota balance in an AOM/DSS-induced CRC mouse model, as confirmed through 16S rRNA sequencing (217). Additionally, another study has shown that caffeic acid phenethyl ester (CAPE), a honeysuckle metabolite of phenolic acid with anti-inflammatory properties, can suppress NLRP3 activation, decrease ROS production, and promote NLRP3 ubiquitination, thus protecting against AOM/DSS-induced colorectal cancer in mice (59). CAPE achieves these effects by inhibiting NLRP3 inflammasome activation and enhancing NLRP3 binding to ubiquitin molecules. Its interaction with CSN5 and Cullin1 further contributes to its antitumor effects. Gut microbiota dysbiosis is a known risk factor for CAC in IBD patients. Panax notoginseng saponins (PNS), a honeysuckle metabolite, has been evaluated in an AOM/DSS mouse model, showing significant relief of colon tumorigenesis and restoration of gut microbiota balance, particularly by increasing akkermansia spp. This suggests that interventions targeting gut microbiota balance could be a promising strategy for preventing CAC in IBD patients (218). These studies reveal that compounds derived from honeysuckle, including dietary polyphenols, ginsenoside compound K, and Panax notoginseng saponins, can effectively manage and treat CAC by modulating gut microbiota and inhibiting inflammatory pathways. These findings highlight the potential of honeysuckle as a natural therapeutic agent in the prevention and treatment of CAC in patients with IBD.

### 6.3 Irritable bowel syndrome

Irritable Bowel Syndrome (IBS) is a common digestive disorder characterized by symptoms such as abdominal pain, bloating, and altered bowel habits (219). Various metabolites found in honeysuckle flowers have shown potential therapeutic effects in managing IBS. For instance, the intervention of quercetin, a polyphenol found in honeysuckle flowers, has demonstrated significant potential in alleviating symptoms of IBS. In post-inflammatory irritable bowel syndrome (PI-IBS) rats, quercetin reduces visceral pain by decreasing 5-HT availability and enterochromaffin cell density, likely through the downregulation of Ngn3 and pdx1 (220). This finding suggests quercetin’s therapeutic potential in managing abdominal hypersensitivity associated with IBS. Similarly, this previous study highlights quercetin’s effectiveness in managing IBS by suppressing inflammatory signaling pathways, a critical factor in the disease’s pathophysiology (221). These anti-inflammatory properties of quercetin improve IBS symptoms. Additionally, luteolin and quercetin, identified in TCM formulations, significantly improve IBS with diarrhea (IBS-D), anxiety, and depression. In a meta-analysis of 25 trials involving 2055 patients, CHM showed higher efficacy in IBS-D treatment [OR = 4.01, 95% CI (2.99, 5.36)] and substantial improvements in depression and anxiety scores, highlighting CHM’s therapeutic potential (222). On the same note, both luteolin and quercetin significantly reduce cytokine expression and secretion, indicating their therapeutic potential for managing IBS and other inflammatory disorders (223). These metabolites also inhibit enzymes like cyclooxygenase and lipoxygenase, contributing to their anti-inflammatory effects and highlighting their broad pharmacological benefits. Besides luteolin and quercetin, CGA, another key metabolite of honeysuckle flowers, significantly alleviates post-infectious irritable bowel syndrome (PI-IBS) by modulating gut microbiota and increasing glycine levels (224). This interaction enhances *bacteroides acidifaciens* extracellular vesicles, which reduce inflammation and intestinal hypersensitivity, thereby maintaining mucosal barrier function and demonstrating a crucial role in PI-IBS pathogenesis. In summary, the numerous bioactive metabolites found in honeysuckle flowers, particularly quercetin and luteolin, show promise in managing IBS symptoms through their anti-inflammatory and antioxidant properties. Their ability to modulate gut microbiota and reduce inflammatory signaling pathways highlights their potential as therapeutic agents in treating IBS and related disorders. Interestingly, a recent clinical trial investigated the potential of *panax ginseng* C.A. Mey. as a treatment for IBS, comparing it with trimebutine in a randomized, double-blind, prospective study (225). The trial involved 24 participants, mostly female (87.5%), who were treated with a daily dose of 300 mg of p. ginseng extract over 60 days. The study found a significant improvement in abdominal pain as assessed by the Likert scale, with participants reporting reductions in pain levels over the course of 8 weeks. This promising result suggests that *panax ginseng*, similar to trimebutine, may play a role in the management of IBS and could warrant further research on its bioactive metabolites in the context of treating IBD or colorectal cancer.

## 7 Conclusion and future perspective

The increasing incidence of IBD, encompassing CD and UC, especially in developing regions, emphasizes the urgent need for novel and effective treatment options. Current therapies often fail to meet patient needs, highlighting the necessity for safer and more efficient alternatives. TCM, particularly *L. japonica*, offers a promising solution due to its minimal toxicity and wide-ranging biological activities. The honeysuckle flower’s bioactive metabolites, such as quercetin, luteolin, cyanidin, chlorogenic acid, caffeic acid, and saponins, have shown significant therapeutic benefits, including anti-inflammatory, immune-regulating, and antioxidant properties. While the honeysuckle bioactive metabolites have beneficial effects, they can also lead to gastrointestinal (GI) irritation or other adverse effects, especially when consumed in high doses. Quercetin, for example, can cause GI discomfort, nausea, or diarrhea in large amounts, particularly in concentrated supplements (226). Luteolin may inhibit pro-inflammatory cytokines; nonetheless, prolonged use could worsen gut dysbiosis in IBD patients, leading to increased inflammation (227). Cyanidin has antioxidant properties but could disrupt gut motility or nutrient absorption in IBD patients, potentially causing nutritional deficiencies (228). Chlorogenic acid and caffeic acid have anti-inflammatory effects, but high doses might increase gut permeability and oxidative stress, worsening IBD (229). Saponins, while immune-modulating, can irritate the GI tract, causing bloating, nausea, and exacerbating gut inflammation in sensitive individuals. These compounds are generally safe at moderate levels, but their interactions with IBD treatments, gut microbiota, and potential adverse effects warrant careful consideration in clinical trials. Research into the mechanisms of *L. japonica* in managing IBD reveals its efficacy in modulating key signaling pathways. The NF-κB pathway, crucial in inflammatory and immune responses, is notably regulated by luteolin and quercetin, which reduce inflammation and oxidative stress. Similarly, the MAPK signaling pathway, which responds to external stimuli, is inhibited by *L. japonica*, demonstrating its role in reducing cytokine levels and intestinal inflammation. The JAK/STAT pathway, vital for managing inflammation and immune responses, is also effectively targeted by honeysuckle metabolites, such as quercetin and chlorogenic acid, reducing cytokine production and improving intestinal barrier integrity. Furthermore, the PI3K and TLR pathways, involved in cell signaling and immune responses, are modulated by *L. japonica*, showcasing its potential to restore gut homeostasis and reduce inflammation. These findings highlight the potential of honeysuckle flower as a comprehensive treatment for IBD. Although this review provides a comprehensive overview of the potential pharmaceutical effects of honeysuckle flower *(L. japonica)* bioactive metabolites in regulating IBD, a significant limitation is the lack of further clinical trial data. Future research, particularly well-designed clinical trials, is essential to fully establish the safety, efficacy, and therapeutic potential of honeysuckle and its bioactive metabolites in the treatment of IBD.
